# Active Learning Accelerates Design and Optimization of Hole-Transporting Materials for Organic Electronics

**DOI:** 10.3389/fchem.2021.800371

**Published:** 2022-01-17

**Authors:** Hadi Abroshan, H. Shaun Kwak, Yuling An, Christopher Brown, Anand Chandrasekaran, Paul Winget, Mathew D. Halls

**Affiliations:** ^1^ Schrödinger, Inc., Portland, OR, United States; ^2^ Schrödinger, Inc., New York, NY, United States; ^3^ Schrödinger, Inc., San Diego, CA, United States

**Keywords:** screening, materials, OLED, optoelectronics, machine learning, HTL

## Abstract

Data-driven methods are receiving increasing attention to accelerate materials design and discovery for organic light-emitting diodes (OLEDs). Machine learning (ML) has enabled high-throughput screening of materials properties to suggest new candidates for organic electronics. However, building reliable predictive ML models requires creating and managing a high volume of data that adequately address the complexity of materials’ chemical space. In this regard, active learning (AL) has emerged as a powerful strategy to efficiently navigate the search space by prioritizing the decision-making process for unexplored data. This approach allows a more systematic mechanism to identify promising candidates by minimizing the number of computations required to explore an extensive materials library with diverse variables and parameters. In this paper, we applied a workflow of AL that accounts for multiple optoelectronic parameters to identify materials candidates for hole-transport layers (HTL) in OLEDs. Results of this work pave the way for efficient screening of materials for organic electronics with superior efficiencies before laborious simulations, synthesis, and device fabrication.

## Introduction

Organic light-emitting diodes (OLEDs) have received significant attention as the most demanded forthcoming display and lighting technology because of their low cost, lightweight, low power consumption, high brightness, and high contrast (Chen et al., 2018; Lee et al., 2019a; Lee et al., 2019b; Abroshan et al., 2020a; Abroshan et al., 2020b; Abroshan et al., 2020c; Abroshan et al., 2020d). Recent developments in OLEDs with flexible panels have opened a new avenue for innovative technologies to fabricate cost-effective large-area, wearables, foldable and shape-fitting displays (Jeong et al., 2020; Song et al., 2020; Yoo et al., 2020). However, further discovery and development are required to improve OLEDs’ efficiency for widespread implementation in optoelectronic devices. In particular, OLED devices showing high quantum efficiency and long lifetimes are in great demand for display and lighting. These optoelectronic devices are composed of multiple layers of thin films, each presenting a different functionality. The stability and the overall performance of the OLEDs are impacted by the properties of the materials in each of the layers, such as chemical structure, morphology, thermal and chemical stability, energy levels, and charge mobility.

Hole transport materials (HTMs) in optoelectronic devices need to be morphologically stable thin films with high hole mobility. Additionally, HTMs should present an appropriate energy level of HOMO (highest occupied molecular orbital) to allow efficient hole injection from the anode into the hole transport layer (HTL) and then the emissive layer (EML) of OLEDs. Furthermore, the energy level of LUMO (lowest unoccupied molecular orbital) of HTMs needs to be at a suitable level to ensure blocking of electron injection from the EML to the HTL. To avoid destroying excitons at the HTL/EML interface, the triplet excited state of the HTMs should be higher, relatively, than that of emitters, blocking triplet energy transfer from emitters to HTMs to confine the excitons in the emissive layer (Fukagawa et al., 2016).

Traditionally, electron-donating moieties with low ionization potential (such as diphenylamine, carbazole, etc.) are utilized to design HTMs with excellent cation radical stability and deliver the charge carrier mobility required for thin films of HTLs. Although the vastly different molecular properties of organic moieties provide an exciting opportunity to design novel HTMs, the enormous space of organic chemistry simultaneously poses a serious challenge in developing novel HTLs with higher performance (Gómez-Bombarelli et al., 2016). Given the numerous potential molecules in the organic space, employing expensive and time-consuming approaches based on chemical intuition and trial-and-error experimentation is practically ineffective. Thus, realizing next-generation OLEDs technologies requires a paradigm change in materials design and development.

Virtual high-throughput screening powered by *ab initio* models has emerged as an exciting alternative to the traditional approach for exploring the molecular space to identify initial hit compounds in materials science and life science research (Hautier et al., 2010; Korotcov et al., 2017; Li et al., 2017; Dubey et al., 2021). However, screening extensive molecular libraries using first principle calculations is expensive, which does not allow efficient evaluation of many materials candidates due to cost.

Recently, active learning (AL) has received much attention to address the challenge of leveraging exhaustive libraries in materials informatics (Vasudevan et al., 2021; LiveDesign Release, 2021; Guyon et al., 2011). In this approach, an adaptive design procedure is employed to provide a feedback loop, which allows the selection of chemical structures from extensive libraries in an efficient and targeted way. This type of machine learning (ML) minimizes the number of expensive and time-consuming physics-based (e.g., DFT) calculations required to identify the best materials candidates and explore the design space for building a reliable ML model. Although AL has been successfully utilized in different fields, such as drug discovery (Eisenstein, 2020) and biology (Pauwels et al., 2014), the application of AL in optoelectronics is in its early stages.

In this paper, we developed an automated workflow to combine AL with density functional theory (DFT) calculations to predict the optoelectronic properties of OLED materials. The proposed AL workflow is designed to efficiently account for multiple optoelectronic parameters to evaluate the overall OLED material’s performance. The AL framework is shown to successfully determine the top candidates from a dataset of all-organic compounds, including ∼9,000 molecules for the hole-transport layer (HTL). This work may offer guidelines to rationally combine atomic-scale simulations with active learning to investigate and design OLED materials with high quantum efficiencies, operational lifetime and thus, be extended to other fields of optoelectronics such as photovoltaics.

The outline of this paper is as follows: The methods and models used in this study are described briefly. The results generated by the AL workflow for the HTL are discussed next. Concluding remarks are offered at the end.

## Materials and Methods

### Datasets

The hole transport materials library examined in this work was built by R-group enumeration based on two groups of chemical structures commonly observed in organic electronic applications. A collection of 38 unique cores from frequently appearing fragments in commercial catalogs and literature (Jhulki and Moorthy, 2018; Swayamprabha et al., 2019) were collected, and then, organic R-groups were generated using a genetic algorithm (Murdock et al., 2015). Following a few cycles of structural enumeration and diversity selection, 8627 representative hole transport candidate materials were chosen to build the library. Structural enumeration steps were designed so that the symmetrically equivalent substitution points will always get the same R-group independently. The dataset of this study are available on request from the corresponding author.

### Active Learning and Multiple Property Optimization

**FIGURE 1:**
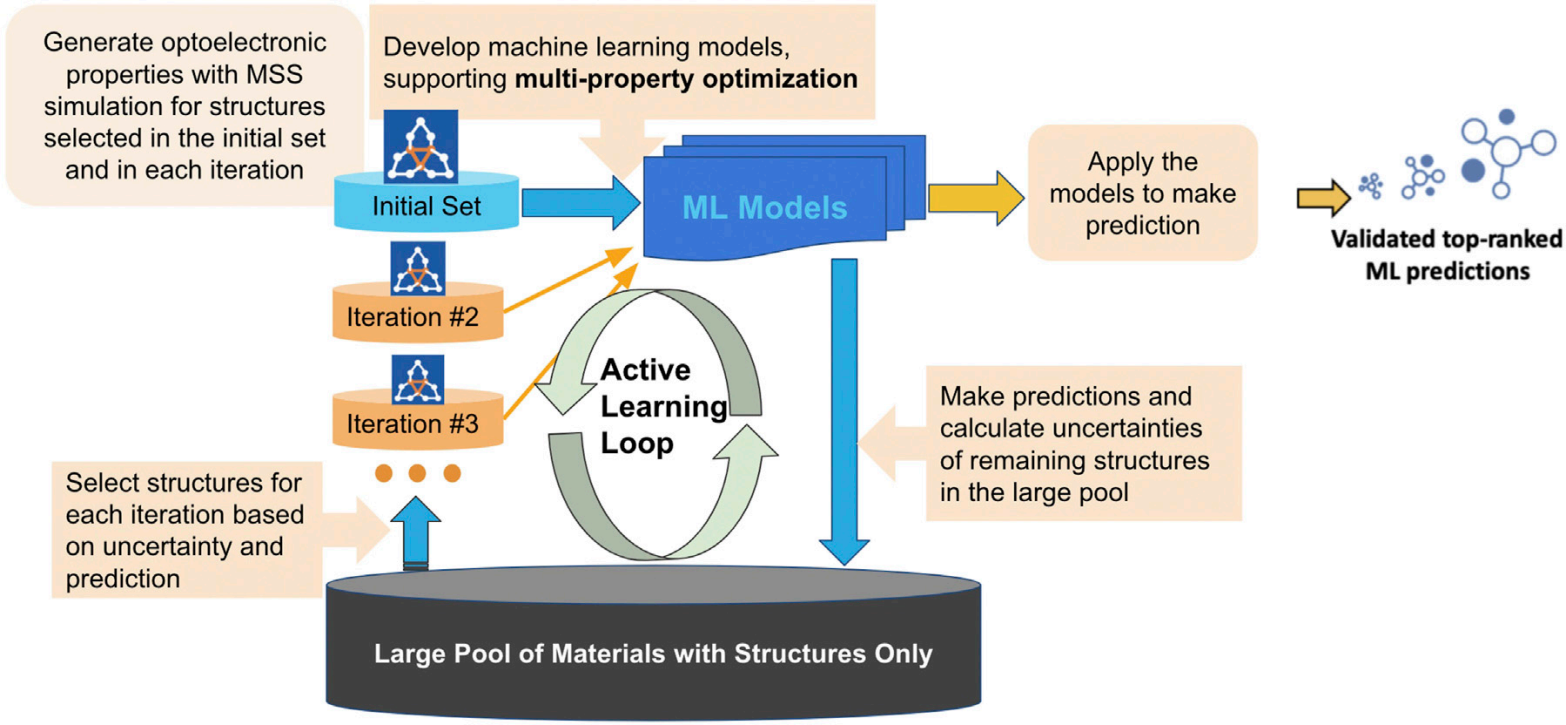
Active Learning workflow for the design and discovery of novel optoelectronic molecules.

Active learning (AL) is employed for intelligent and iterative identification of promising materials candidates within a large dataset (Figure 1). In this approach, machine learning and DFT are synergistically combined. In the framework of AL, the predicted value with associated uncertainty is considered to decide what structures to be added in each iteration, aiming to improve the model’s performance in the next iteration. In this process, machine learning algorithms and featurization (descriptor generation) are both important. In the present study, we used 200 cheminformatic descriptors and a combination of circular fingerprints with topological torsion fingerprints (RDKit, 2020) to featurize the chemical structures. These fingerprints and descriptors generated on the initial subset of structures are given as vectors to an algorithm based on Random Forest (Pedregosa et al., 2011). Bayesian Optimization is employed to tune the hyperparameters of the model. In each iteration, a trained model is applied for making predictions on the remaining materials in the dataset. We note that for the latter materials, no DFT data is available. Since it is important to consider multiple properties in material discovery, we used a multiple property optimization (MPO) score (Kwak et al., 2016; Schrödinger, 2020) to scale and combine multiple properties into a single score. Here, MPO score is desired to be maximized for obtaining the desired property profile.

The MPO scheme used in this work translates each property data to a dimensionless value between 0 and 1, using a logistic function (see the equation below), whose shape can be further tuned by defining fixed boundaries. In our study, we used 0.8 and 0.2 in the converted score for each property as thresholds for “good” (0.8 or above) and “bad” (0.2 or below).

$$\;f\left(x\right)\;=\;\frac{1}{1+e^{-b\left(x-a\right)}}$$ (1)

If the property needs to be maximized (identified as a “higher better” mode with b > 0) or to be minimized (identified as a “lower better” mode with b < 0), a single logistic function can be used to set up the desirability score per property space. If the target property is sandwiched between the less-desired property domains (identified as a “middle good” mode), we break down the property space into two spaces and solve for the two logistic functions - “higher better” mode on the left and “lower better” mode on the right - that are facing each other in line symmetry at the target property.

Once the logistic function *f*(*x*) is solved for each property space, *f*(*x*) becomes the desirability score for a compound that has the property *x*. The MPO score is then generated by taking the geometric mean of the desirability scores.

In addition to predicting the MPO score, we also calculate the uncertainties/standard deviations of the Random Forest model using the infinitesimal jackknife variance method (Wager et al., 2014). When selecting the next rounds of molecules to be added to the training set, we devised the “expected improvement score,” which is the combination of predicted property values in the form of a MPO score and the uncertainty measure of the molecule. The utilization of the expected improvement function ensures the optimal tradeoff between exploration and exploitation to avoid being trapped in a local minimum of chemical space, and molecules with high uncertainties are included in the training set to improve machine learning model performance. Once DFT calculations are performed on the selected molecules, the training set is augmented with the newly calculated properties, and the active learning cycle is repeated.

All DFT calculations within the AL framework were automatically performed using B3LYP functional with the MIDIXL basis sets available via the Jaguar package developed by Schrӧdinger Inc. (Schrödinger Release, 2021; Bochevarov et al., 2013).

### Featurization and Machine Learning Model Training

A 90%/10% training and test set split is used. The hyperparameters of the Random Forest estimator are tuned using Bayesian Optimization (and are thus different for every run). The three hyperparameters that were tuned were the number of trees in the forest (n_estimators), within a range of 1–250, the minimum number of samples required to split an internal node (min_samples_split), within a range of 2–25, and the number of features to consider as a fraction of the total number of features when looking for the best split (max_features), within a range of 0.1–0.999.

200 Rdkit descriptors were used capturing various physicochemical properties such as molecular weight, fraction of aromatic atoms, topological polar surface area (area). A detailed description of the descriptors can be found on the Rdkit website (RDKit, 2020). In addition to the 200 descriptors, circular (also known as Morgan) fingerprints and topological torsion fingerprints were used. The circular (bit-based) fingerprints had a radius of 2 and described the presence/absence of certain substructures/fragments around atoms in a molecule. Topological torsion fingerprints are a linear sequence of four consecutively bonded non-hydrogen atoms, each described by its atomic type, the number of non-hydrogen branches attached to it, and its number of pi electron pairs (Marcus, 1993).

### Reorganization Energies

Hole-transport layers (HTL) of OLED devices are desired to present high rates of hole transfer. According to the semiclassical Marcus equation (Nilakantan et al., 1987):

$$k_{h}=\frac{2\pi }{\hbar }{\left|t\right|}^{2}\frac{1}{\sqrt{4\pi \lambda_{h}k_{B}T}}\exp\left(-\frac{{\left(\lambda_{h}+△G{}^{\circ}\right)}^{2}}{4\lambda_{h}k_{B}T}\right)$$ (2)

for estimating charge hopping rates, the hole transport rates *(k_h_)* are inversely proportional to the charge transport’s corresponding reorganization energies $\left(\lambda_{h}\right)$ . Thus, it is desired to minimize $\lambda_{h}$ to improve the hole transport rates in the materials. In the Marcus equation, *ħ*, *k*_B_, and *T* denote the reduced Planck constant, Boltzmann constant, and temperature. The top candidates with low $\lambda_{h}$ can be further studied by evaluating electronic couplings (*t*) between neighboring molecules and the associated change in free energy (Δ*G* ^o^) for the charge transports. In the framework of AL, the reorganization energies for a hole $\left(\lambda_{h}\right)$ are automatically calculated employing the potential energy surface (PES) calculations discussed in detail elsewhere (Samanta et al., 2017). Here, hole reorganization energies were calculated as:

$$\lambda_{h}\;=\;\left(E_{0}^{+}\;-\;E_{+}^{+}\right)+\;\left(E_{+}^{0}\;-\;E_{0}^{0}\right),$$ (3)

where E_0_^+^ denotes the energy of the cation at the optimized geometry of the neutral molecule; E_+_^0^ is the energy of the neutral molecule at the optimized geometry of the cation; E_+_^+^ and E_0_^0^ are the energy of the cation and neutral molecule at their corresponding optimized geometries.

### Oxidation Potential

**FIGURE 2:**
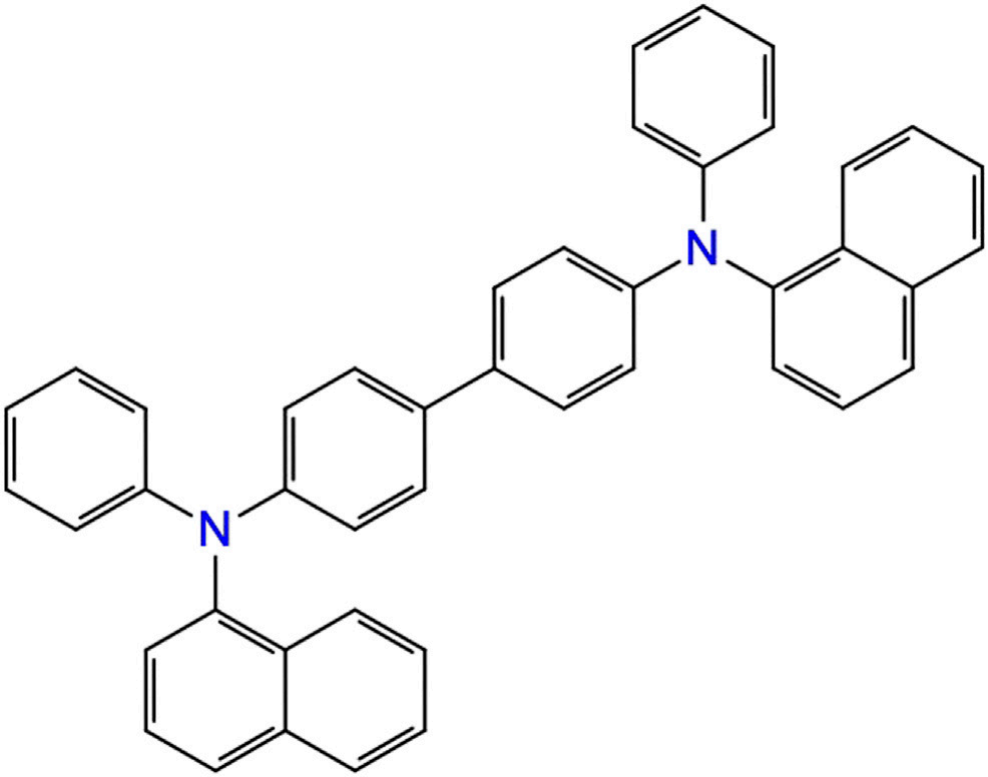
Chemical structure of NBP, which is used as a reference for the oxidation potential.

For hole transport materials, in addition to the low $\lambda_{h}$ to improve the rates of hole transport, the HOMO energy (oxidation potential, *V*_Oxidation_) is required at an appropriate level to ensure a low energy barrier for hole injection from the anode into the emissive layer. As a reference, we considered 4,4′-Bis [N-(1-naphthyl)-N-phenylamino]biphenyl (NPB), which is one of the most commonly used as HTL compounds in OLEDs, see Figure 2.

Previous studies have shown that using a relatively inexpensive level of theory (B3LYP/MIDI!) combined with empirical corrections are able to predict orbital energies in good agreement with experimental measurements (Kondakova et al., 2008; Shukla et al., 2009). Here, the empirical corrections are applied to produce the Koopman redox potentials using the raw density functional orbital energies:

$$V_{\mathrm{Oxidation}}=-17.50\cdot E_{\mathrm{HOMO},DFT}\left(eV\right)-2.17$$ (4)

The oxidation potential of NPB using Koopmans’ method is found to be 0.88 V, which is in acceptable agreement with the experimental measurements of 0.75 V (Jia et al., 2005).

As discussed above, the reduction potential (LUMO energy) and triplet state energies of HTMs also contribute to the overall performance of OLED devices. These properties can also be included in the calculations within the AL framework. For simplicity and speed, in this paper, we exclude these properties to estimate the MPO scores of the materials.

## Results and Discussions

We applied an automated workflow that efficiently integrates active learning (AL) and density functional theory (DFT) calculations for the high-throughput screening of ∼9,000 compounds. The materials’ performance for hole transport layer (HTL) is examined by screening their hole reorganization energies $\left(\lambda_{h}\right)$ and oxidation potentials. Here, $\lambda_{h}$ is desired to be minimized (<0.1 eV) for fast hole transport rate between neighboring molecules in HTL. Materials with $\lambda_{h}$ > 0.3 eV are considered as not suitable for HTL. Furthermore, we used the calculated oxidation potential of NPB (0.88 V) as a target value with inner and outer tolerances of 0.15 and 0.3 V, respectively. Materials with oxidation potentials of 0.88 ± 0.15 (i.e., inner tolerance) V are considered to be good candidates for HTL. If oxidation potential of a material is estimated to be outside of 0.88 ± 0.30 (i.e., outer tolerance) V range, the material is considered to be not suitable for HTL. In this study, the significance (i.e., weight) of hole reorganization energies and oxidation potentials to calculate multiple property optimization (MPO) scores of materials is considered to be the same. The size of the training set for AL was increased from 50 to 550 molecules in 10 iterations. In this study, maximum simulation subjobs were set to be 50 (i.e., 50 CPUs), and the calculations were completed within ∼85 h.

**FIGURE 3:**
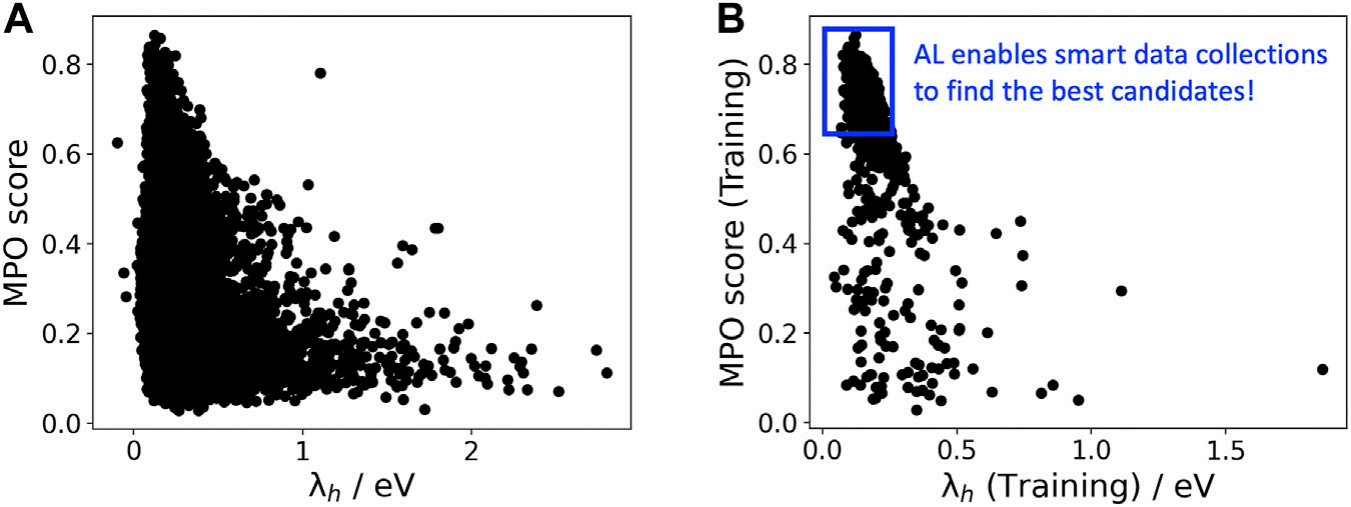
**(A)** MPO score of all materials in the HTL dataset and **(B)** those used in the training set as a function of $\lambda_{h}$ .

Figure 3A displays MPO scores for the HTL dataset estimated by AL as a function of hole reorganization energies that are separately calculated for all the materials. This figure indicates that there are many materials in the dataset with desired low hole reorganization energies but are not suitable for HTL due to their improper oxidation potentials. This result shows that the AL workflow efficiently accounts for multiple parameters to evaluate the optoelectronic performance of the materials.

Figure 3B presents MPO scores of the materials used in the training dataset of AL, demonstrating that the feedback loop in the AL workflow efficiently guides the data collection as the size of the training set increases. This approach provides a time-efficient route to find promising materials candidates across a huge chemical space.

**FIGURE 4:**
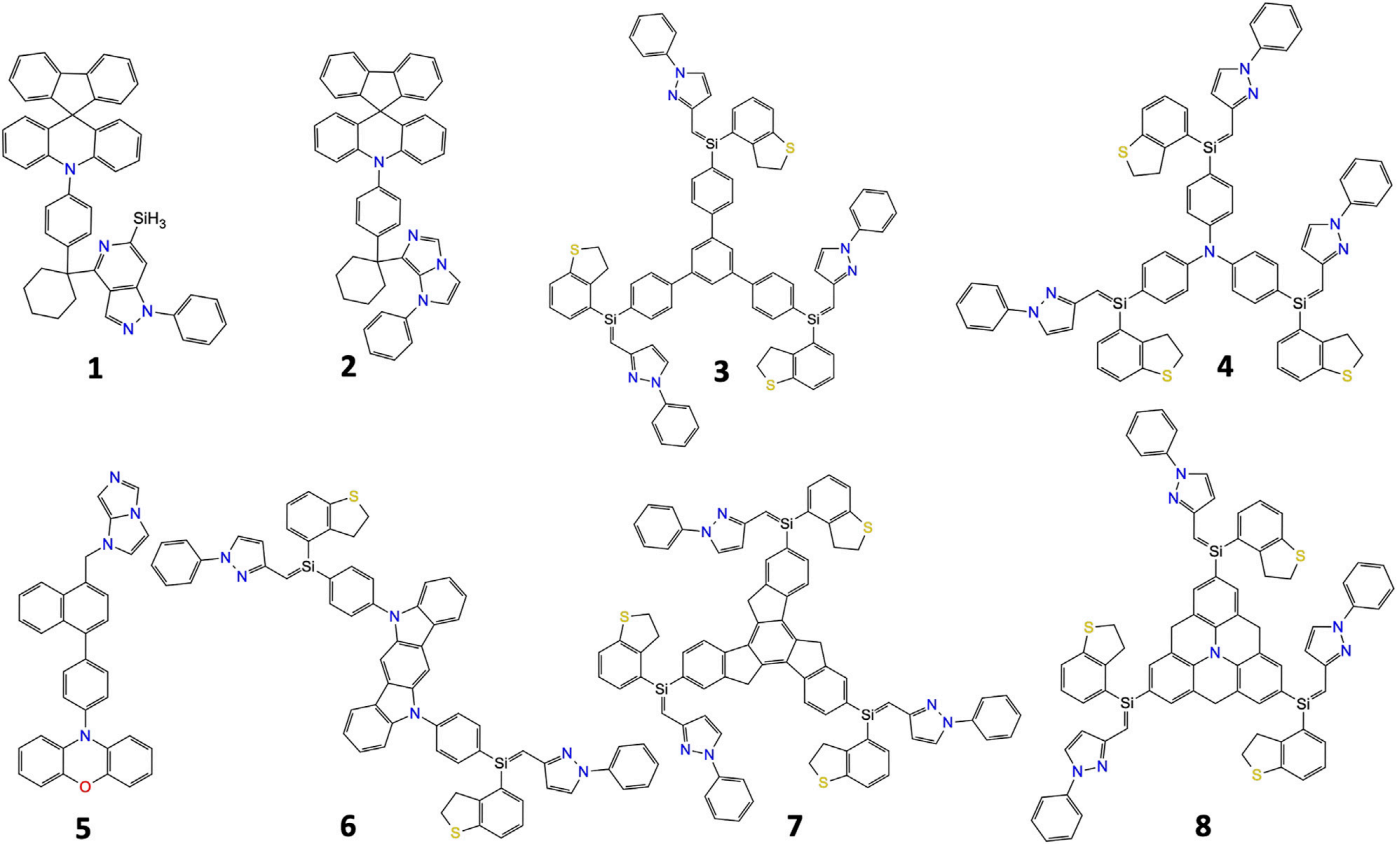
Some of the materials candidates with top MPO scores for the hole transport layer.

Examples of top candidates with the highest MPO scores in the dataset of hole transport layer materials are presented in Figure 4. Results demonstrate that the AL workflow could successfully select candidates with chemical cores that are known to offer good performance in hole transport layers such as triphenylbenzene-, triphenylamine-, carbazole-, and spiro-based structures. Although the AL framework provides a unique platform to filter out top candidates based on reorganization energies and oxidation potentials, the selected candidates need further investigations to estimate their electronic couplings between neighboring molecules, hole transport rates in thin films, LUMO levels and triplet states energies. For example, we calculated LUMO and triplet state energies of materials shown in Figure 4. Results are summarized in Table 1. Calculated LUMO level of molecules 1, 3-8 are moderately deeper than that for NPB, while it is significantly up-shifted for molecule 2. This result indicates that the latter molecule may successfully block electron injection to the HTL. Triplet state energy of molecules 1, 2 and 5 is calculated to be higher than that of NPB (2.46 eV), illustrating that the use of the latter HTMs is expected to minimize triplet energy transfer at HTL/EML interface, thus excitons are confined in the emissive layer.

**TABLE 1:** Calculated LUMO and triplet state energy (T_1_) for some of the materials shown in Figure 3 and NPB. All energies are given in eV.

Material	LUMO	T_1_
1	−2.48	2.84
2	−1.89	3.10
3	−2.75	1.35
4	−2.76	1.35
5	−2.62	2.75
6	−2.64	1.34
7	−2.74	1.33
8	2.70	1.25
NPB	−2.30	2.46

Additionally, the materials for HTL are required to present good thermal stability in the amorphous state to avoid a significant change in the hole mobility under operating conditions. Here, molecular dynamics simulations and neural-network-based machine learning can provide insights into glass transition temperature (*T*_g_), determining their thermal stability. Of note, one of the core structures that is frequently selected by the AL workflow presents a spiro configuration. The spiro-core structure is deemed to remarkably increase the *T*_g_ value of materials (Swayamprabha et al., 2019), and thus enhance their thermal stability, making them suitable materials with high efficiency and a long operational lifetime for OLED devices.

**FIGURE 5:**
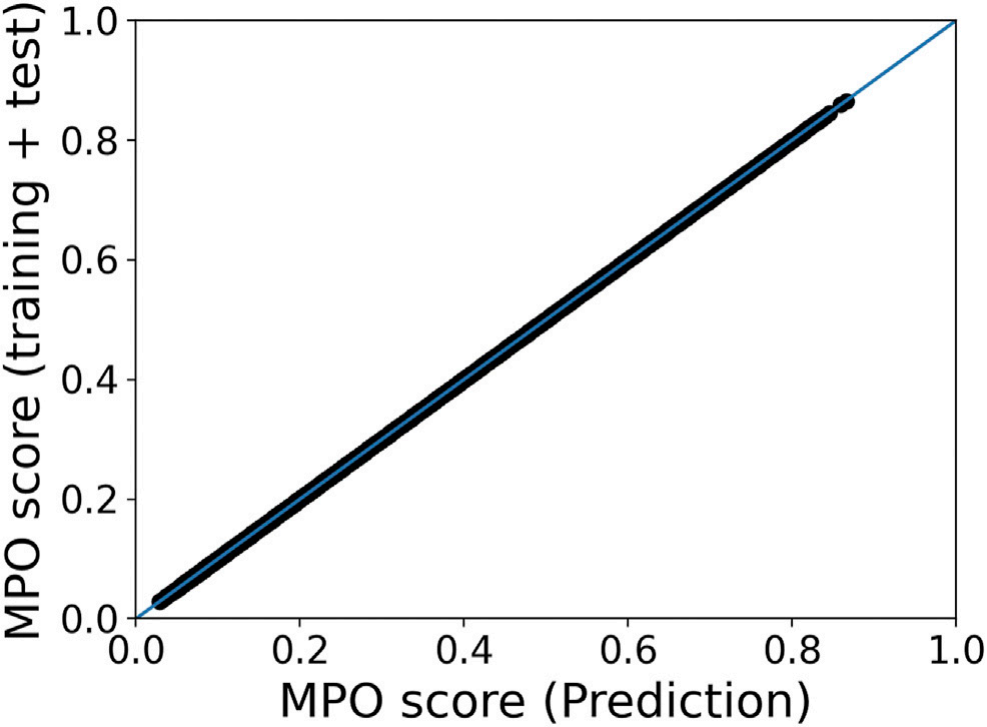
Linear correlation of MPO scores of materials in the HTL dataset obtained during the training of the ML model in the framework of AL and the scores estimated by using the ML model thus generated for the same dataset.

It is noteworthy that the AL module developed in the Maestro package provides ML models that can be used later to predict MPO scores of other materials relevant to the dataset for which ML models are optimized. To further validate the AL workflow, we used the ML model generated for the same dataset of hole transport layer materials and predicted MPO scores of the materials. Figure 5 displays an acceptable linear correlation between the MPO scores calculated during training of the ML model by active learning and those scores predicted using the optimized ML model. This result points to the reproducibility of the MPO scores, indicating that the AL workflow is robust and holds promise for practical materials informatics.

## Conclusion

We present an active learning workflow for accelerated design and optimization of novel OLED materials. The fully automated module efficiently combines atomic-scale calculations at the level of DFT with active learning ML to reliably predict the optoelectronic properties of OLED materials. This study used a dataset to identify top materials candidates for hole transport using multiple property optimization (MPO) scores for hole transport materials. Results showed that the workflow enables a fast, high-throughput screening of materials, filtering out the best molecules to be further examined for practical applications. Here, for a dataset of ∼9,000 molecules, the AL workflow determined the top candidates for HTL by evaluating 550 molecules in 10 iterations using DFT calculations. Performing DFT calculations for all of the 9,000 molecules in the dataset would increase the computational cost by a factor of 15 × ‘s versus the Active Learning (AL) workflow. This study describes a robust framework to accurately link molecular and chemical descriptors to OLED materials properties. The rapid screening of datasets by AL enables improved understanding of structure-function relationships for systematic design and application of OLED materials with higher efficiency.

## Data Availability

The original contributions presented in the study are included in the article/Supplementary Material, further inquiries can be directed to the corresponding author.
